# Harmonic Distortion Measurements for Non-Destructive Evaluation of Steel Strips

**DOI:** 10.3390/s25092769

**Published:** 2025-04-27

**Authors:** Anastassios Skarlatos, Roberto Miorelli

**Affiliations:** Université Paris-Saclay, CEA, List, F-91120 Palaiseau, France

**Keywords:** magnetic measurements, magnetic sensors, material evaluation, harmonic distortion, hysteresis, coercive field, mechanical properties, microstructure

## Abstract

There is an increasing demand for better and more accurate non-destructive evaluation and testing techniques for ferromagnetic materials, in particular, steel structures, which span diverse industrial sectors. Among the available methods, the measurement of the harmonic content and the corresponding distortion due to the material non-linearity is one of the most widespread approaches, with significant implications in the steel industry. Although these approaches have been in use for several decades with considerable success, crucial aspects, including the sensor design, the fine-tuning of the method, and, most importantly, the processing and interpretation of the acquired signals, remain, to a certain degree, empirical. Meanwhile, significant progress in simulation and data processing techniques has been reported in recent years, whose potential in the further development of the existing measuring technology cannot be overstated. In view of this rapidly changing landscape, the present review article aims to provide an overview of the state-of-the-art of the harmonic distortion measurement technique from the perspective of the recent advances in simulation and inversion methods.

## 1. Introduction

Non-destructive material evaluation methods serve two basic purposes: i. retrieving characteristic material properties, and ii. assessing the degree of degradation with respect to a nominal state in a non-destructive way, i.e., without altering the structural integrity. They are introduced to replace, or at least minimise, expensive destructive tests, i.e., experiments involving local piece deformations (as in the case of the hardness test) or the removal of small specimens for further laboratory examination. It is well-understood that the latter family of methods is subjected to a number of practical and economical restrictions, which exclude the possibility of in situ examination.

When speaking of ferromagnetic materials, a key property that non-destructive measurements rely on is the strong correlation of the magnetic field with the material microstructure. Since magnetic field is an easily experimentally accessible quantity, one can understand that its measurement in the vicinity of the piece can deliver rich information about the state of the material. There are different available magnetic measurements, each one focussing one or more features of the magnetisation curve, depending on the material characteristics that one needs to reveal. Hristoforou, Ktena, Vourna, et al. used differential permeability measurements for the nondestructive assessment of residual stresses [1,2,3]. Residual stresses are also well correlated with the incremental permeability, as was demonstrated by Uchimotto, Duscharne et al. [4,5,6,7]. The magnetic Barkhausen noise (MBN) proves to be a very sensitive tool for hardening detection and characterisation, as is shown in a number of works [8,9,10,11,12,13,14,15]. Martinez-de-Guerrenu used a number of different identifiers derived from hysteresis curves, such as the coercive field, the remanence, and the hysteresis losses, to monitor the recovery process during annealing [16,17,18].

Many of the cited works concern laboratory measurements involving specially prepared specimens, which encounter a number of serious limitations when coming to in situ non-destructive measurements. An important step towards a systematic and robust exploitation of different magnetic identifiers suitable for outdoor/industrial environments was taken by Dobmann and coworkers during the 1980s. Their pioneering contributions resulted in the introduction of the 3MA (Micromagnetic-, Multiparameter-, Microstructure-, and Stress-Analysis) measuring concept [19,20,21], which was subsequently turned into a commercial device [22]. The 3MA system combines three different magnetic measurements, namely, harmonic distortion, incremental permeability, and MBN with a classical eddy-current measurement, which are then supplied as inputs to a dedicated regressor to provide predictions for different properties like yield and tensile strength, hardness, etc. Similar commercial solutions, albeit ones focused on specific measurements, are now offered by other companies such as the PropertyMon [23] or the IMPOC system [24].

The usefulness of such measurements has been demonstrated after years of operation in more than one steel production line and a number of associated research projects [25,26,27,28,29,30,31]. Despite the very satisfactory results so far, the rationale behind these technologies remains very pragmatic, heavily relying on the existence of calibration curves. There is a certain need for more quantitative results that will benefit from the recent advances in the domain of numerical simulation and signal processing.

In the present review article, we are interested in the particular case of the harmonic distortion measurement, which we try to examine from the above-mentioned perspective. Starting with the general mathematical statement of the characterisation problem and the definition of the inputs and outputs, as well as the involved mathematical operators, we proceed to a presentation of the underlying physical concepts and the typical experimental setups used for these kinds of measurements. This introductory section is complemented by a brief presentation of the three commercial implementations mentioned above: the 3MA, the PropertyMon, and its predecessor, the HACOM system.

In the second section, the focus shifts to the study of the material law. Different parametric models for the calculation of the magnetic hysteresis are discussed and classified according the the number of input parameters. This piece of information allows one to have an image of the problem dimensionality, which is an important aspect when we try to construct inverse models. Afterwards, the spectral decomposition of the operator is discussed, in an effort to give a qualitative understanding of how the hysteresis parameters are linked to the harmonic content. At the end of this section, the harmonic distortion variable is introduced, which is a quantity that provides an alternative figure of merit for magnetic materials.

The third section is dedicated to the processing of the measured signals. To facilitate understanding, some simple analytical and semi-analytical models for the calculation of the field response with the three considered setups are examined, followed by a detailed discussion of the model inversion.

The main ideas discussed in the article are numerically illustrated by means of a representative example. At the end of the paper, a short account of the correlation with the microstructure and the mechanical properties that are the main quantities of interest for real-world applications are given.

## 2. The Harmonic Distortion Measurement

### 2.1. Formal Statement of the Characterisation Problem

A ferromagnetic material is characterised by its magnetisation, which is a function of the externally applied magnetic field H and the material microstructure, the latter being mathematically expressed by a set of appropriate parameters p:

(1) M=M(H;p).

The dimension of p and the physical meaning of the involved parameters depend upon the model used to describe M. For this reason, p is intentionally kept unspecified until the introduction of the hysteresis’ explicit mathematical expression. All we need to know at this level is that p provides a complete characterisation of the material.

**Figure 1:**
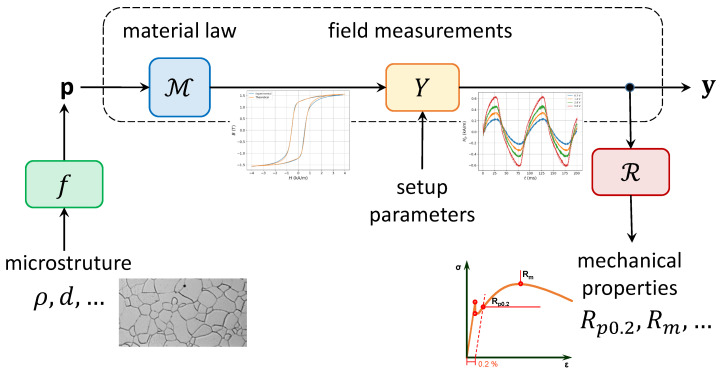
Conceptual image of a magnetic characterisation task. The material parameters p, controlled by the underlying microstructural features, are indirectly determined via dedicated field measurements y and can be obtained by the inversion of the total transfer operator MY. The electromagnetic measurements can be also used for indirect assessment of the mechanical properties via specially designed regressors.

The conceptual image of the magnetic characterisation measurement is schematically depicted in Figure 1. Our primary task is to determine p in terms of a set of suitable electromagnetic observables, herein referred to as y. In other words, we seek to establish the relation y↦p. The measurement process itself can be understood as the concatenation of two operators: the material operator M, which translates the material parameters p to the magnetisation function (which is nothing more than the numerical evaluation of (1) under the given operational conditions of the setup),

(2) M:p↦M

and the measurement operator, which associates the material magnetisation state to the observables,

(3) Y:M↦y.

Y is a linear operator that only depends on the setup geometry, and its expression can be calculated by solving the underlying electromagnetic problem for the magnetic field around the piece and thereupon calculating its convolution with the transfer function of the sensor. This is the task of the electromagnetic solver, which is, in a general case, a numerical solver such as the finite element method (FEM) or the finite difference/integration method (FDM/FIT). In the case of simple geometries, this calculation can be also carried out analytically. Some standard approximate analytical solutions for three typical setups are given in a later section for completeness.

The determination of the material operators p can be thus formally obtained by calculating the inverse operator

(4) $$p={\left(\mathrm{YM}\right)}^{-1}y=M^{-1}Y^{-1}y.$$

In the particular case of harmonic measurements, one usually does not proceed to the end of the chain by inverting YM but is usually satisfied with the information hidden in the spectral information of the measurement; otherwise stated, one works with the ratio of the higher harmonics to the fundamental one.

We assume that a sinusoidal excitation of radial frequency $\omega_{0}$ is exerted upon the specimen. There are two main excitation modes: either by controlling the magnetic field or the mean magnetic flux that passes through the specimen cross-section (in the case of uniaxial configurations). Whatever the excitation mode, all remaining (non-imposed) magnetic variables will contain the basic frequency with all the higher harmonics, a direct result of the field interaction with the non-linear material operator. Thereupon, the resulting expression for the magnetic field reads

(5) $$H\left(t;p\right)=\sum_{n=0}^{\infty }H_{2n+1}\left(H_{0},p\right)\sin\left(\omega_{2n+1}t+\theta_{n}\right)$$

with $\omega_{2n+1}=\left(2n+1\right)\omega_{0}$. The fact that only odd harmonics appear in (5) is a well-known experimental fact and reflects the point symmetry property of the hysteresis. We shall comme to this point in the following section. Note that, in the special case of an *H*-imposed excitation, $H_{2n+1}=0,\forall n>0$.

The corresponding time signals for the magnetisation and the measurements will admit similar decompositions, namely,

(6) $$M\left(t;p\right)=\sum_{n=0}^{\infty }M_{2n+1}\sin\left(\omega_{2n+1}t+\phi_{n}\right)$$

and

(7) $$y\left(t\right)=\sum_{n=0}^{\infty }y_{2n+1}\sin\left(\omega_{2n+1}t+\psi_{n}\right).$$

Specific attention should be drawn here that the ratios between the harmonics of the measured quantities are not identical to those of the material operator and, hence, the latter should not be confused with the former. In fact, for a rigorous extraction of the material information, one needs to remove the spectral deformation introduced by the transfer function of the measurement disposition. This de-convolution operation is the task of the inverse electromagnetic solver:

(8) $$\begin{array}{cc} M_{2n+1} & =\left|Y^{-1}\left[y_{2n+1}e^{i\psi_{2n+1}}\right]\right| \end{array}$$

(9) $$\begin{array}{cc} \phi_{2n+1} & =\arg\left\{Y^{-1}\left[y_{2n+1}e^{i\psi_{2n+1}}\right]\right\}. \end{array}$$

Coming back to Figure 1, one can also discern two side branches. The lower-right branch relates the mechanical parameters of the material with the experimental observables y. Lacking a rigorous mathematical framework, the usual way to perform this prediction is via regression, which is denoted in the figure via the action of the R operator. The construction of the appropriate regression operator, however, is not a simple task since it necessitates a number of (destructive) calibration measurements, which are not always available.

The lower-left branch associates the input model parameters p with actual microstructural features like the grain size and the dislocation density. Also, here, a complete theory is missing at the moment. Instead, a number of empirical formulas that establish certain links between the microstructure and basic hysteresis characteristics exist in the literature, which also require extensive calibration. Following the complete chain from the lower-left to the lower-right permits (at least theoretically) the establishment of an indirect assessment of the destructively determined mechanical parameters with the underlying microstructure, which is of strong interest in many industrial applications. More on this point is discussed in the last part of this article.

### 2.2. Measurement Principle, Basic Setups and Experimental Observables

**Figure 2:**
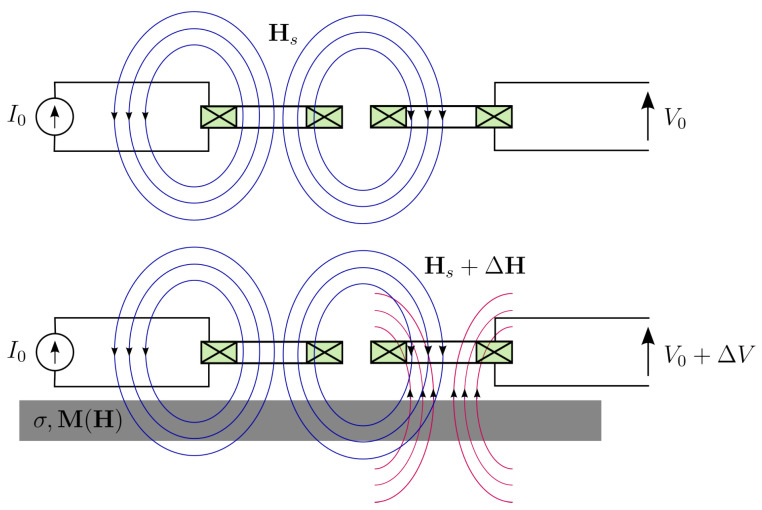
Principle of magnetic measurements. (**Upper**) An induction coil is used to establish the source (primary) magnetic field $H_{s}$ in the region of the tested specimen. (**Lower**) $H_{s}$ interacts with the specimen and creates a reaction field, which is measured by a second coil (or, equivalently, by a magnetic sensor).

Magnetic methods for the characterisation of ferromagnetic materials exploit the interaction between an inductor (or a system of these) and the tested specimen, which is brought in close interaction. The working principle is schematically explained in Figure 2.

According to Ampère’s equation, the inductor will induce a magnetic field in the surrounding area, including the piece itself:

(10) $$\nabla \times H_{s}=J$$

where J is the current density in the inductor windings and $H_{s}$ is the induced source field.

The piece will interact with $H_{s}$, with the resulting total magnetic field after the interaction H being given by the relation

(11) $$\nabla \times \nabla \times \left(H-H_{s}\right)=-\sigma \frac{dB}{dt}$$

where σ is the electrical conductivity of the medium and B stands for the magnetic induction, which is locally related with the magnetic field via the magnetic constitutive relation:

(12) $$B\left(H\right)=\mu_{0}\left[H+M(H)\right]$$

$\mu_{0}$ being the magnetic permeability of the free space and M(H) the magnetisation of the medium. The latter contains all the physics of the material and, thereupon, its microstructural information. In non-magnetic regions like the air M=0, and (12) reduces to a simple linear relation.

The solution to (11), together with the constitutive relations in (12) and the boundary conditions at the piece interfaces, will provide the sought H and B values inside the piece and the air. The solution of the Maxwell equations in the presence of a ferromagnetic medium is a difficult problem, which is the subject of the whole research area of computational electromagnetism. For this reason, we cannot proceed to a detailed discussion in the limited space of this article. A number of representative articles are given instead (recall once more that the literature is huge and the following list does not claim by any means exhaustiveness): [32,33,34,35,36,37,38,39,40,41,42,43,44]. In Section 4.2, a semi-analytical solution is sketched for the case of an axisymmetric problem, which is meant to serve as example solution for these kinds of problems.

From the above discussion, it is inferred that the total field in the vicinity of the material contains indirect information about the material microstructure through the employment of M(H). This information can be retrieved by reading the field response at some point close to the piece. This can be achieved in two ways, either by directly measuring the magnetic field using a magnetic sensor like the Hall effect, giant magnetoresistance/magnetoimpedance (GMR/GMI), flux-gate, etc., or by measuring the time derivative of the magnetic flux density using the exciting or a secondary inductor. In the second case, our observable will be the electromotive force (EMF) at the inductor’s terminals $V_{emf}$, which is given by Faraday’s law:

(13) $$V_{enf}=-N\frac{d\Phi }{dt}$$

where *N* is the number of turns and Φ is the magnetic flux crossing the inductor cross-section, namely,

(14) $$\Phi =\int_{A}B\cdot dA.$$

**Figure 3:**
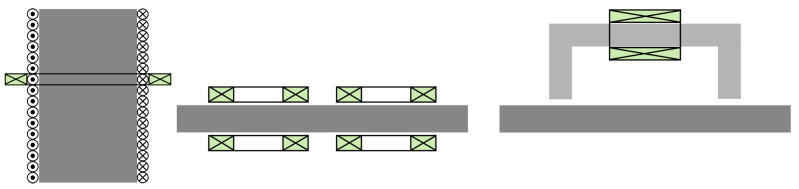
Basic setups for quasi-uniaxial magnetic measurements. (**Left**) Solenoid. (**Centre**) Open magnetic cicruit. (**Right**) Closed magnetic circuit.

The typical setups for conducting this type of measurement are illustrated in Figure 3. In the first-from-the-left configuration, a homogeneous magnetic field is induced in the tested specimen by means of a long solenoid, and the flux variation is measured using a secondary coaxial to the solenoid coil. This is the simplest configuration, which suffers, however, from a major drawback: its application is restricted to narrow specimens (bars or ribbons) and, hence, this can be considered as a destructive technique. A very popular variation of the solenoid setup is the Epstein frame [45].

The second configuration involves a number of induction coils with a specific phase relation between them, which serves in guiding the magnetic field lines inside the tested specimen. It is suitable for testing planar specimens of arbitrary lateral dimensions, which is the major advantage of this method. In cases where only one side is accessible, the method is still applicable, yet less efficient, since the absence of flux concentrators (ferrite cores) and the less-favorable coil arrangement result in a significant number of stray fields. These types of arrangements are also referred to as open-circuit setups. An industrial realisation can be found under the name of the HACOM and its successor, the ProperyMon device [23].

The third configuration involves a closed magnetic circuit in the form of a ferromagnetic or ferrite yoke. For an even better magnetic coupling to the tested piece, a second yoke can be added to the opposite surface. This variation is known as a single-sheet (or single-strip, in the case of narrow specimens) tester [45]. It is the configuration that implements the 3MA device, another popular industrial device for the magnetic testing of ferromagnetic pieces [22].

### 2.3. Industrial Realisations: 3MA, HACOM, and PropertyMon Systems

In the previous paragraph, mention was made to three popular commercial devices that have been successfully used for the quality control of the steel strips in production lines via the in-line monitoring of the magnetic properties: the 3MA, the HACOM, and the PropertyMon system. All three devices perform harmonic distortion measurements (alongside others) and are capable of predicting properties like the yield $R_{p0.2}$ and tensile strength $R_{m}$, using integrated multi-parameter regression tools.

**Figure 4:**
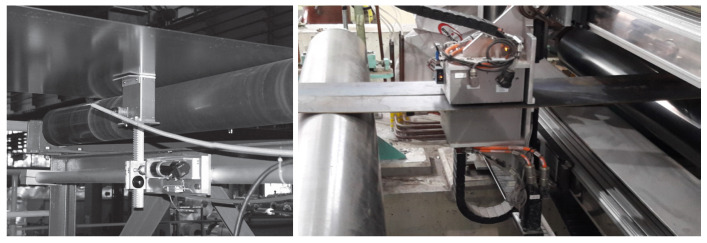
Examples of 3MA and PropertyMon plant installations for the in-line monitoring of steel strips. (**Left**) 3MA probe (image reproduced from [25]). (**Right**) PropertyMon heads (image courtesy of Primetals).

The 3MA device was originally based on the contributions of Dobmann et al. during the 1980s and has undergone a number of further developments/improvements thereafter; it is currently commercialised by the IZFP institute in Saarbrücken. It offers four types of magnetic measurements, namely, harmonic analysis, magnetic Barkhausen noise (MBN), incremental permeability, and multi-frequency eddy-current measurements. Its setup involves a closed magnetic circuit approach, combined with secondary coils for performing the inductive measurements and a magnetic sensor for the acquisition of the tangential magnetic field component. It is delivered with an integrated software module, which permits data analysis and regression tools for the prediction of mechanical properties like $R_{p0.2},R_{m}$, hardness, etc. An example of 3MA installation for the monitoring of steel quality in a production line is given in Figure 4.

The HACOM instrument is an industrial realisation of the second configuration shown in Figure 3, involving an intelligent arrangement of air-cored coils. Similarly, with the 3MA, it is combined with a dedicated software for performing acquisition, data analytics, and regression for the estimation of the key mechanical/technological properties of the tested specimen.

PropertyMon can be considered as the successor of HACOM (which is no longer commercially available), based on the same principle with its predecessor, yet offering essential improvements in terms of hardware and software tools. PropertyMon is commercialised by Primetals Technologies Austria GmbH, in Linz, Austria. A typical plant integration of the product is given in Figure 4.

## 3. Magnetic Properties

### 3.1. Parametric Models for the Description of Magnetic Hysteresis

The magnetisation function M(H,p) has been introduced in (1) without giving details of its form or its properties. In this section, we are interested in specifying the properties of the magnetisation function and providing approximate expressions for its calculation.

In its general form, M is a non-linear, hysteretic, and anisotropic function. Since, however, working with a fully vectorised version of the magnetisation is complicated (and, for many practical situations, unnecessary), it is much more convenient to work with the special case of scalar hysteresis, i.e., the magnetic law only applies to the amplitudes of the magnetic variables:

(15) M=M(H,p)

with M=|M| and H=|H|. This simplification is a good approximation in situations where the magnetic field is predominantly in a specific direction. This is certainly the case in single-strip configurations and applies equally well in open-circuit setups such as the PropertyMon, where the magnetic field is intentionally guided parallelly to the specimen surface.

There is a plethora of magnetic hysteresis models in the literature, such as the Preisach model [46], the Jiles–Atherton model [47,48], the Stoner–Wohlfarth model [46], the Hauser (or energetic) model [49], the Mel’gui model [50], and the dry-friction model [51], just to mention some of the most well-known among them. One may also mention the Rayleigh model [52], but, since this model is only applicable in the weak-field region, it has been deliberately omitted from the above list. It can be, however, used as a constitute of hybrid formulations for the improvement of the calculations at the low-field domain, where most of the above models behave rather poorly [53].

An important question that has to be answered when dealing with characterisation is the minimum number of parameters that one needs to capture the basic hysteresis features. The larger the number of parameters the model relies on, the better the fit with the experimental data, but the more difficult its identification becomes (recall that the parameter estimations are achieved via the solution of an inverse problem).

**Figure 5:**
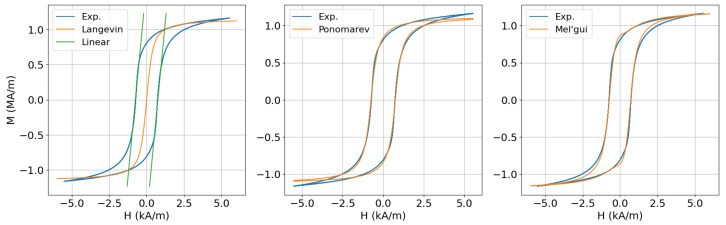
Comparison of different hysteresis approximations with increasing numbers of parameters: (**Left**) two-, (**Centre**) three-, and (**Right**) five-parameter models.

The absolute minimum of parameters that one needs, when dealing with a non-linear model, is two. These can be the slope at zero field $\chi_{0}$ (initial susceptibility) and the high-field asymptote (magnetisation at saturation) $M_{s}$, or the coercive field $H_{c}$ and the corresponding susceptibility value $\chi_{c}$. Both approximations provide a partial description of the full hysteresis curve, which can be useful when we are only interested in a particular subdomain. In particular, a parametrisation based on $\left(\chi_{0},M_{s}\right)$ describes the high-field regime well, which is dominated by reversible domain rotations, whereas the second parametrisation based on the local information around the coercive field $\left(H_{c},\chi_{c}\right)$ is useful when working with step hysteresis loops or when we are primarily interested in the behaviour around the coercive point. This approximation is also known in mechanics as the backlash model, and this term is also used herein. The two approximations are illustrated in Figure 5(left) for the case of dual-phase (DP) steel. The measured maximum curve of the material is also given for comparison. For this particular illustration, the Langevin model has been used for the high-field approximation, but other models can perform equally well. The explicit expression of the Langevin expression reads

(16) $$M\left(H\right)=M_{s}\left[\coth\left(\frac{H}{a}\right)-\frac{a}{H}\right]$$

where the *a* parameter is related to the initial susceptibility via the relation $a=M_{s}/3\chi_{0}$.

In order to be able to reconstruct the closed loop, one needs at least one additional parameter that provides the information of the loop width, expressed by the coercive field. A simple expression for the hysteresis loop was proposed by Ponomarev in the 1980s [54]:

(17) $$M^{\pm }\left(H,H_{r}\right)=\frac{2M_{s}}{\pi }\arctan\left(\frac{H\mp H_{c}}{H_{0}}\right)\pm \frac{\delta M(H_{r})}{2}$$

where

(18) $$\delta M\left(H_{r}\right)=\frac{2M_{s}}{\pi }\left\{\arctan\left(\frac{H_{r}+H_{c}}{H_{0}}\right)-\arctan\left(\frac{H_{r}-H_{c}}{H_{0}}\right)\right\}.$$

$M_{s}$ is the saturation magnetisation, $H_{c}$ the coercivity, $H_{0}$ is a parameter describing the squareness of the hysteresis loop, and $H_{r}$ stands for the magnetic field amplitude at the reversal point. The negative/positive sign applies to the ascending/descending branch.

The $H_{0}$ parameter can be expressed by the more familiar remanent magnetisation $M_{R}$ by setting H=0 in (17) and solving for $H_{0}$, which yields

(19) $$H_{0}=\frac{H_{c}}{\tan(\frac{\pi }{2}\frac{M_{R}}{M_{s}})}.$$

In addition to its striking simplicity, the Ponomarev model succeeds in delivering a fairly good description of the experimental data for a large family of soft industrial steels, where the irreversible magnetisation mechanisms due to wall movement are predominant. The comparison of the model prediction with our example DP steel is shown in Figure 5(center). It is worth noting that the given model is also interesting because, although originally introduced as a heuristic expression, it can be derived from first principles under certain assumptions, as shown in [55]. In the same paper, it is also shown that the skewness parameter $H_{s}$ is linked to the material disorder. The derivation of (17) is given in Appendix A for completeness.

A generalisation of Ponomarev’s expression was proposed by Mel’gui in [50], which introduces a supplementary empirical term in order to improve the model accuracy in the high-field region, where domain rotational mechanisms become important. This improvement comes, however, at the cost of two additional parameters. The Mel’gui expression reads

(20) $$\begin{array}{cc} M^{\pm }\left(H,H_{r}\right)=\chi_{0}\frac{H_{c}^{2}}{H^{2}+H_{c}^{2}}H\; & \mp \frac{H_{r}^{2}}{H_{r}^{2}+kH_{c}^{2}}M_{p}^{\pm }\left(H,H_{r}\right) \end{array}$$

where $M_{p}^{\pm }$ is the Ponomarev expression given in (17). The two additional parameters introduced in Melgui’s experssion are the initial susceptibility $\chi_{0}$ and the weighting coefficient *k*, defined as

(21) $$k=\frac{M_{s}}{\pi }\frac{\arctan\left[2\tan(\frac{\pi }{2}\frac{M_{R}}{M_{s}})\right]}{M_{c}-\chi_{0}\frac{H_{c}}{2}}-1.$$

$H_{c}$ stands for the coercive field, $M_{R}$ is the remanence, and $M_{c}$ is defined as the virgin curve value that corresponds to the coercive field, namely, $M_{c}=M_{in}\left(H_{c}\right)$. The model performance is illustrated in Figure 5(right). We can easily observe the better fit for the higher field values.

A summary of the discussed models and their tuning parameters is given Table 1.

### 3.2. Spectral Analysis of the Hysteresis Operator

After having introduced the parametric model for the description of the hysteresis operator, the next question that we need to address is how the model parameters are associated to the spectrum of the magnetisation function (or its derivative, the differential susceptibility), which is the property of our concern.

In order to proceed with this discussion, we need to select a working hysteresis model. The model of choice has to be as simple as possible but, an the same time, representative of the main material features. Ponomarev’s model makes a good candidate given its simple form, the restricted number of parameters, and the very good approximation of real hysteretic curves that it provides.

Although, the magnetisation spectrum can be easily computed numerically, it is instructive to try to derive approximate analytic relations, involving explicit expressions of the material parameters $M_{s},H_{c}$, and $H_{c}$. This can be achieved by Taylor series development, which yields the following expression for the lowest harmonics, assuming a sinusoidal excitation of the form $H\left(t\right)=H_{m}\sin\omega_{0}t$:

(22) $$M_{2n+1}\approx \frac{2M_{s}}{\pi }\sum_{k=0}^{5}c_{k,n}{\left(\frac{H_{c}}{H_{0}}\right)}^{k}e^{-i\left(2n+1\right)\omega_{0}t}.$$

The derivation of (22) and the explicit expressions of the series coefficients are given in Appendix B.

**Figure 6:**
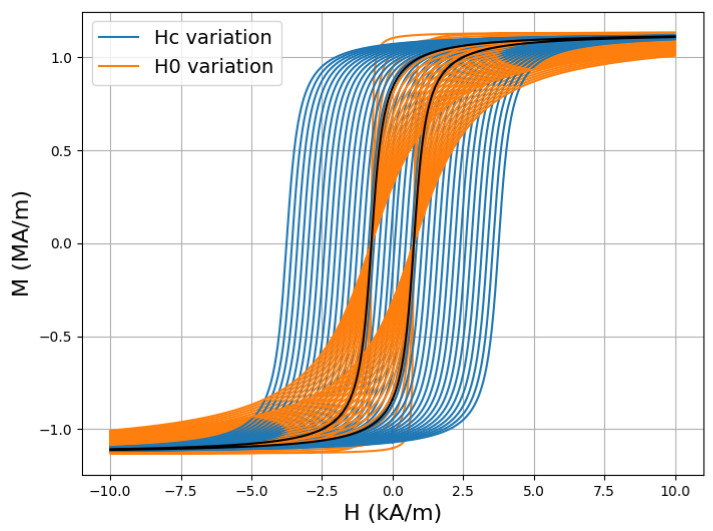
Hysteresis curves obtained for varying $H_{c}$ and $H_{0}$. The initial curve obtained using the nominal parameters is drawn in black for comparison.

The results of the previous analysis can be complemented by numerical studies with the Ponomarev model in Expression (17) for variable $H_{c}$ and $H_{0}$ and by calculating the harmonic coefficients of the resulting magnetisation signal via Fourier transform. As study material, we consider the same IF steel grade used for the studies in [55]. The material parameters are obtained by the identification of the experimental curves, following the standard procedure, and they are summarised in Table 2. We then let $H_{c}$ and $H_{0}$ vary in the interval 10–500% with respect to their nominal value. The resulting bunch of curves obtained with the considered values are demonstrated in Figure 6.

**Figure 7:**
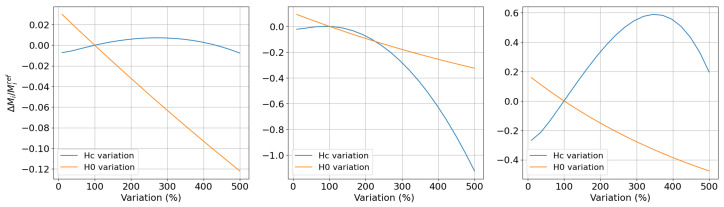
Relative variation of the 1st, 3rd, and 5th harmonics corresponding to the hysteresis curves of Figure 6. Note that the curves corresponding to $H_{c}$ variations have been multiplied by a factor of 100 for visualisation purposes.

The relative variation of the first three harmonics with respect to their value obtained for the original curve is illustrated in Figure 7. From a comparison of the curves, it turns out that $H_{c}$ variations result in a stronger variation of the higher harmonics than the ones when varying $H_{0}$. In other words, the sensitivity of the harmonic measurements with respect to the coercive field is stronger that that for the form factor $H_{0}$. In this particular example, the curves corresponding to $H_{c}$ variation have been multiplied by a factor of 100.

### 3.3. The Harmonic Distortion Identifier K

Instead of directly using the harmonic amplitudes $M_{2n+1}$, many practitioners prefer working with two derived indicators: the coercive field $H_{c}$ and the harmonic distortion factor *K*, defined as the ratio of the mean of the higher harmonic amplitudes $M_{2n+1}$ to that of the basic harmonic $M_{1}$:

(23) $$K=\sqrt{\frac{\sum_{n=1}^{\infty }\left|M_{2n+1}^{2}\right|}{|M_{1}{|}^{2}}}.$$

The advantage of using the harmonic distortion identifier is that it directly integrates the information from several harmonics, thus offering better robustness to noise. Assuming that we have estimated $H_{c}$ independently, the knowledge of *K* together with $H_{c}$ is theoretically sufficient to identify the normalised Ponomarev’s model (and, indirectly, the material itself).

In the previous paragraph, the dependency of the first three harmonics from the $H_{c}$ and $H_{0}$ parameters has been demonstrated both analytically and numerically. From the dimensionality of the parameters space, it turns out that one needs to measure at least two harmonics for the identification of the $H_{c},H_{0}$ parameters. We can, therefore, infer that $H_{c}$, *K* deliver sufficient information for the reconstruction of the normalised hysteresis curve.

On the contrary, since $M_{s}$ is canceled out as a common factor in all terms in (23), its determination requires an additional independent measurement. The simplest choice is with proceed to an eddy-current measurement, which provides us the complementary information of the initial magnetic susceptibility $\chi_{0}$ (under the assumption that there is zero remanence at the measurement point). Using the Langevin expression at zero-field in (16), we easily derive the relation initial susceptibility $\chi_{0}=M_{s}/3a$. The *a* parameter can be estimated by fitting the normalised Langevin curve to the high-field part of the (also normalised) Ponomarev model, which leaves us with only one unknown parameter: $M_{s}$.

## 4. Measurement Processing and Inversion

### 4.1. Semi-Analytical Approximations for the Inverse Operator

Given the complexity of the field problem, the calculation of the transfer function Y is carried out, in most cases, numerically. Nevertheless, reasonable analytical (or semi-analytical) approximations can be obtained for simplified versions of the setups in Figure 3. The interest in such approximations is not only academic. Fast approximate solutions can be used to give a coarse qualitative image of the setup response, and they can be combined with minimisation algorithms for the solution of the inverse problem, which tend to be numerically cumbersome when using numerical solutions.

Stating with the solenoid, the calculation of both Y and $Y^{-1}$ is very straightforward. The variable of interest here is the voltage at the leads of the receiving coil:

(24) $$y\left(t\right)=V\left(t\right)=-\mu_{0}N_{c}A\frac{d}{dt}\left(N_{e}I+M\right)$$

where $n_{e}$ are the linear density of the solenoid windings (in turns/m), $\mu_{0}$ is the magnetic permeability of air, $A,N_{c}$ are the cross-section and the number of turns in the receiver coil, and *I* the excitation current. Equation (24) can be trivially inverted, giving

(25) $$M\left(t\right)=-\frac{1}{\mu_{0}N_{c}A}\int y\left(t\right)dt-n_{e}I\left(t\right).$$

In the case of the closed magnetic circuit in Figure 3, a simple coarse approximation can be obtained by using the well-known magnetic circuit model. The very low magnetic reluctivity of the yoke allows us to assume that the magnetic flux is preferentially contained inside the yoke volume, with only a weak proportion of the magnetic streamlines straying away. Furthermore, since the yoke core has a very low conductivity, the eddy-current effect can be ignored, which implies that the magnetic field distribution throughout the yoke cross-section is nearly homogeneous.

**Figure 8:**
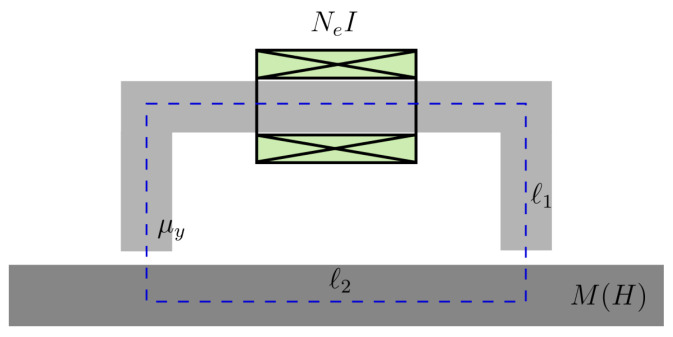
Working model for the magnetic circuit approach.

At a first order, we may also assume that the same approximations also hold inside the tested specimen, though less accurately than in the yoke. We may additionally assume that the field is approximately constant along the yoke and along the specimen, which is equivalent with stating the the demagnetisation field due to the geometry boundaries is negligible. These kinds of models, where the spatial variation of the involved quantities is discarded, are classified in the literature as 0D or lumped models. The above-described assumptions of the working model are schematically depicted in Figure 8.

The application of Ampère’s law along the dashed integration path of Figure 8 yields

(26) $$H_{1}\ell_{1}+H_{2}\ell_{1}=N_{e}I$$

where $H_{1},H_{2}$ are the magnetic field in the yoke and the specimen, respectively, $\ell_{1},\ell_{2}$ are the corresponding path lengths, $N_{e}$ is the number of turns in the excitation coil, and *I* the feed current.

It is also reasonable to assume that the M(H) curve of the yoke is very narrow, with low coercivity $H_{c}^{\left(y\right)}$ and high saturation field, which allows us to approximate it with a linear magnetic material of high relative permeability $\mu_{y}$. This assumption is very close to reality for many commercial yokes. One can thus write

(27) $$B_{1}\left(H\right)\approx \mu_{y}H_{1}.$$

Imposing the continuity of the magnetic flux density across the yoke poles, we end up with the expression

(28) $$M_{2}\left(t\right)=-\left(1+\frac{\mu_{y}}{\mu_{0}}\frac{\ell_{2}}{\ell_{1}}\right)H_{2}\left(t\right)+\frac{\mu_{y}}{\mu_{0}}\ell_{1}^{-1}N_{e}I.$$

The solution is the common point of (28) and $M_{2}\left(H_{2}\right)$, which is obtained by iteration.

Our observable is the tangential magnetic field $H_{2}$, which is measured by means of a magnetic field sensor (Hall element, GMR, etc.). Assuming a linear response function for the sensor, we can write

(29) $$y\left(t\right)=KH_{2}\left(t\right).$$

Both simplified models examined above share the common assumption that the entire magnetic flux passes through the piece. The effect of the piece conductivity has been also tacitly ignored, resulting in a homogeneous field in the piece cross-section. Should the fringing effect at the piece interfaces be non-negligible or should the conductivity play an important role, the field distribution become more complicated, which requires the use of more sophisticated approaches. Even in such situations, however, semi-analytical solutions, where the field is developed in a series, remain tangible. This is the case, for example, in the open-circuit setup shown in Figure 3 if a single pair of coils is involved.

The development of the semi-analytical solution for this case is described in [43]. Here, we simply sketch the main ideas of the approach in order to give the coarse image.

The magnetic field in this case is 2D, which makes it more convenient to work with a uniaxial magnetic potential instead of the magnetic field itself. The common choice is the magnetic vector potential, which, in the case of an axisymmetrical problem, reduces to a single-component potential along the azimuth direction ϕ, namely, $A=Ae_{\phi }$, with $e_{\phi }$ standing for the corresponding unit vector. The magnetic potential is related to the magnetic induction via the relation

(30) B=∇×A.

Assuming a harmonic excitation of the form $e^{i\omega_{0}t}$, the resulting state equation for the *n*th harmonic of the magnetic potential is the diffusion equation, which in the cylindrical coordinates system (ρ,ϕ,z) reads:

(31) $$\left(\nabla^{2}-\frac{1}{\rho^{2}}\right)A_{n}-i\mu_{0}\sigma \omega_{n}A_{n}=-\mu_{0}J\delta_{n,1}-\mu_{0}e_{\phi }\cdot \nabla \times M_{n},\;n=1,3,\ldots$$

where $\mu_{0}$ is the air permeability, $\delta_{n,1}$ is the Kronecker delta, and *J* is the current density in the coil. Note that *J* is zero everywhere except inside the coil. M stands for the material magnetisation, and it is only nonzero inside the plate. Although it is not a priori known, it stands in the right-hand side of (31) since it acts as a magnetic source. Its value is estimated via the successive solution of (31) by requiring the fulfillment of the material law (12), according to the standard fixed-point technique. (The reader may notice here that M is not a uniaxial field in the 2D case considered here, which raises questions about the adequateness of the scalar hysteresis approach adopted throughout this article. In fact, it can be shown that the normal to the plate $H_{z}$ component is much weaker that the tangential one $H_{\rho }$, which makes the field solution inside the plate quasi-uniaxial. This is a peculiarity of the special coil arrangement in this setup).

Due to the odd symmetry of the field with respect to the *z*-axis, we set the z=0 point to be in the middle of the plate and we truncate the domain by considering only the z≥0 axis. To be consistent with the problem symmetry, we also need to assume a perfect magnetic conductor condition at z=0. In order to simplify the spectral expressions, we assume an artificial boundary at a certain radial distance from the coil $\rho_{L}$. This truncation is justified by the fact that the field is rapidly decreasing as we move away from the source. The type of termination condition is irrelevant. For simplicity, we assume a perfect magnetic wall at $\rho =\rho_{L}$.

The solution for the magnetic potential in the air region above the plate reads

(32) $$A_{n}^{\left(1\right)}\left(\rho ,z\right)=\sum_{\ell =1}^{\infty }\left[C_{n\ell }^{\left(s\right)}e^{\kappa_{\ell }\left(z-d/2\right)}+D_{n\ell }^{\left(d\right)}e^{-\kappa_{\ell }\left(z-d/2\right)}\right]J_{1}\left(\kappa_{\ell }\rho \right)$$

where $C_{n\ell }^{\left(s\right)}$ and $D_{n\ell }^{\left(d\right)}$ are the series coefficients for the source term and reflection from the plate and $J_{1}\left(\kappa_{\ell }\rho \right)$ are the first-order Bessel functions. The eigenvalues $\kappa_{\ell }$ are determined by the boundary condition at the truncation surface:

(33) $$J_{1}\left(\kappa_{\ell }\rho_{L}\right)=0.$$

The solution is more complicated inside the plate since, now, the right-hand side of (31) is nonzero. The series development for the homogeneous Helmholtz equations needs to be complemented with a second term that takes care of the magnetisation term. We can thus write

(34) $$\begin{array}{cc} A_{n}^{\left(2\right)}\left(\rho ,z\right) & =\sum_{\ell =1}^{\infty }J_{1}\left(\kappa_{\ell }\rho \right)\left\{C_{n\ell }^{\left(c\right)}\sinh\left(v_{n,\ell }z\right)+\sum_{p=1}^{\infty }c_{n\ell p}\sin\left(\alpha_{p}z\right)+c_{n\ell 0}z\right\} \end{array}$$

with

(35) $$v_{n\ell }^{2}=i\omega_{n}\mu \sigma +\kappa_{\ell }^{2}$$

and the $\alpha_{p}$ eigenvalues being given by

(36) $$\alpha_{p}=2p\pi /d.$$

The $c_{n\ell p}$ coefficients stand for the special solution of (31), and they are calculated by projecting the right-hand side to the development basis:

(37) $$c_{n\ell p}=\frac{4}{\left(i\omega_{n}\mu \sigma +\kappa_{\ell }^{2}+\alpha_{p}^{2}\right)E_{\ell }^{2}d}\int_{0}^{\rho_{L}}\int_{0}^{d/2}\left(e_{\phi }\cdot \nabla \times I\right)J_{1}\left(\kappa_{\ell }\rho \right)\sin\left(\alpha_{p}z\right)\rho d\rho dz$$

where $E_{\ell }=\rho_{L}J_{0}\left(\kappa_{\ell }\rho_{L}\right)$ is the normalisation coefficient of the Bessel function basis. $c_{n\ell 0}$ are obtained by observing (37) at z=d/2, namely,

(38) $$c_{n\ell 0}=\frac{2}{\left(i\omega_{n}\mu \sigma +\kappa_{\ell }^{2}\right)E_{\ell }^{2}}\int_{0}^{\rho_{L}}\left(e_{\phi }\cdot \nabla \times I\right)J_{1}\left(\kappa_{\ell }\rho \right)\rho d\rho dz.$$

The $D_{n\ell }^{\left(d\right)}$, $C_{n\ell }^{\left(c\right)}$ coefficients stand for the homogeneous solution, and they are determined by applying the continuity relations at the piece boundary. The final relations read

(39) $$\begin{array}{cc} D_{n\ell }^{\left(d\right)} & =-RC_{\ell }^{\left(s\right)}-\frac{1+R}{2\mu_{r}\kappa_{\ell }}\left[\sum_{p=1}^{\infty }{\left(-1\right)}^{n}\alpha_{n}c_{n\ell p}+\left(1-\frac{1-R}{1+R}\frac{\mu_{r}\kappa_{\ell }d}{2}\right)c_{n\ell 0}\right] \end{array}$$

and

(40) $$\begin{array}{c} C_{n\ell }^{\left(c\right)}=TC_{n\ell }^{\left(s\right)}+\frac{T}{2\mu_{r}\kappa_{\ell }}\left[\sum_{p=1}^{\infty }{\left(-1\right)}^{n}\alpha_{n}c_{n\ell p}+\left(1+\frac{\mu_{r}\kappa_{\ell }d}{2}\right)c_{n\ell 0}\right] \end{array}$$

where *R* and *T* are Fresnel’s reflection and the transmission coefficient, respectively:

(41) $$R=\frac{v_{n\ell }\cosh\left(v_{n\ell }d/2\right)-\mu_{r}\kappa_{\ell }\sinh\left(v_{\ell }d/2\right)}{v_{n\ell }\cosh\left(v_{n\ell }d/2\right)+\mu_{r}\kappa_{\ell }\sinh\left(v_{\ell }d/2\right)}$$

(42) $$T=\frac{2\mu_{r}\kappa_{\ell }}{v_{n\ell }\cosh\left(v_{n\ell }d/2\right)+\mu_{r}\kappa_{\ell }\sinh\left(v_{\ell }d/2\right)}.$$

The source coefficients $C_{\ell }^{\left(s\right)}$ are obtained by solving the field problem with the coil in the area, and, therefore, they depend only upon the coil geometry and the excitation current. Their expression for the region below the coil and the plate (we are usually interested only in the field in the immediate neighbourhood of the plate interface; similar expressions that hold for the domains beside and over the coil can be readily obtained, yet their explicit expressions are not provided here for brevity—the interested reader can refer to [56]) is as follows:

(43) $$\begin{array}{cc} C_{\ell }^{\left(s\right)} & =-4\mu_{0}\iota_{0}\sinh\left(\frac{\kappa_{n}l}{2}\right)\frac{\chi (\kappa_{n}\rho_{in},\kappa_{n}\rho_{out})}{\kappa_{n}^{5}{\left[\rho_{L}J_{1}\left(\kappa_{n}\rho_{L}\right)\right]}^{2}}J_{m}\left(\kappa_{n}\rho_{0}\right)e^{-\kappa_{n}\left(z_{0}-d/2\right)} \end{array}$$

where $\rho_{in,out}$ stand for the coil inner and outer radius, respectively, *l* is the coil thickness, and $\iota_{0}$ is the current density across its cross-section. The χ function stems from the integration over the coil cross-section and it has a closed-form expression in terms of Struve functions $H_{n}\left(x\right)$: $\chi \left(x_{1},x_{2}\right)=\frac{\pi }{2}{\left[xJ_{0}\left(x\right)H_{1}\left(x\right)-xJ_{1}\left(x\right)H_{0}\left(x\right)\right]}_{x_{1}}^{x_{2}}$.

The measurement in this case is either the voltage variation at the terminals of the coils or the tangential magnetic field at a specific observation position $\left(\rho_{o},z_{o}\right)$. In the second case, we can write, for the *n*th harmonic of the response,

(44) $$\begin{array}{cc} y_{n} & =-\frac{K}{\mu_{0}}\sum_{\ell =1}^{\infty }\kappa_{\ell }\left[C_{n\ell }^{\left(s\right)}e^{\kappa_{\ell }\left(z_{o}-d/2\right)}-D_{n\ell }^{\left(d\right)}e^{-\kappa_{\ell }\left(z_{o}-d/2\right)}\right]J_{1}\left(\kappa_{\ell }\rho_{o}\right). \end{array}$$

### 4.2. Direct Extraction of the Coercive Field from the Magnetic Field

The direct measurement of the magnetic flux density B is not possible in setups involving open or closed magnetic circuits. This inhibits the direct measurement of the coercive field $H_{c}$. Alternative approaches for an approximate estimation of $H_{c}$ were proposed by Dobmann et al. [57,58] and also by Fillon et al. [59], where they demonstrated, either experimentally or via simulation, that $H_{c}$ is closely related to the zeros of the second time derivative of the tangential magnetic field. This point also proves to be correlated with the cross-over point of the higher magnetic field component (i.e., the signal we obtain by subtracting the basic harmonic).

A good theoretical justification of the first observation is given in [57]. Let us consider the definition relation of the differential susceptibility, which, using the chain rule, can be written as the quotient of the time derivatives of *M* and *H*:

(45) $$\chi_{d}=\frac{dM}{dH}=\frac{dM/dt}{dH/dt}$$

The time derivative of the susceptibility reads

(46) $$\frac{d\chi_{d}}{dt}=\frac{\frac{d^{2}M}{dt^{2}}\frac{dH}{dt}-\frac{dM}{dt}\frac{d^{2}H}{dt^{2}}}{{\left(\frac{dH}{dt}\right)}^{2}}.$$

Given that the susceptibility becomes a maximum at the coercive field, and given that the magnetic field is stationary only at the turning points of the hysteresis, i.e., at the maximum of the excitations, we can safely assume that the denominator is nonzero in the region of interest. We can hence conclude that

(47) $${\left[\frac{d^{2}M}{dt^{2}}\frac{dH}{dt}-\frac{dM}{dt}\frac{d^{2}H}{dt^{2}}\right]}_{H=H_{c}}=0.$$

We also know that, at the coercive point, the Bloch wall mobility also reaches a maximum. In the simple of a two-domain system separated by a single wall, it can be shown should be present here that the magnetisation rate is related with the velocity of the wall via the relation [60]:

(48) $$\frac{dM}{dt}=2M_{s}\frac{<v>}{L}$$

where <v> is the mean wall velocity and *L* is its length. From (48), and by assuming that the rate of the mean wall velocity is stationary at the coercive point d<v>/dt=0, we can state that the coercive field is an inflection point for the magnetisation. Let $t_{c}$ be the time instance when reaching the coercive field, i.e., $H\left(t_{c}\right)=H_{c}$. Then,

(49) $${\left.\frac{d^{2}M}{dt^{2}}\right|}_{t=t_{c}}=0.$$

Combined with (47), the last condition yields

(50) $${\left.\frac{d^{2}H}{dt^{2}}\right|}_{t=t_{c}}=0.$$

In other words, the coercive point is an inflection point for the magnetic field as well.

Conditions (49) and (50) are naturally satisfied in case of an induction-driven system: the magnetic flux density *B* is the experimentally controlled variable (since the external magnetic field is the physical intensive variable that is accessible to the experimentalist, an external closed-loop system is necessary for the realisation of a *B*-driven system). Indeed, from the constitutive equation, we have

(51) $$\frac{d^{2}B}{dt^{2}}=\mu_{0}\left(\frac{d^{2}H}{dt^{2}}+\frac{d^{2}M}{dt^{2}}\right).$$

B=0 at $t_{c}$ by definition. For a sinusoidal magnetic induction, it follows that the second derivative is also zero at $t_{c}$, and, taking (47) into account, we obtain

(52) $${\left[\left(\frac{dH}{dt}+\frac{dM}{dt}\right)\left(\frac{d^{2}H}{dt^{2}}\right)\right]}_{t=t_{c}}=0.$$

The first term is nonzero, which naturally yields (50). It is clear that Condition (50) cannot be fulfilled when the magnetic field is externally imposed (e.g., for a solenoid excitation). In that case, we must conclude that (49) cannot hold and that the mean wall velocity is not at its exact maximum there. In other cases between the two extremes of pure sinusoidal *B* or *H*, both fields dispose higher harmonics, and we can thus conclude that (49) and (50) are approximately fulfilled [57,59]. These are the cases where there is an *M*-*H* coupling due to the presence of a demagnetising field caused by the piece boundaries.

The second approximation for the coercive field is based on the location of the zeros of the higher harmonic components. It is known from theory that the discontinuity of the normal magnetisation component at the piece interfaces acts as an effective magnetic surface-charge density:

(53) $$\sigma_{m}=n\cdot M\approx n\cdot B.$$

at the coercive point B=0, which implies that $\sigma_{m}\approx 0$. Thereupon, we can conclude that the demagnetisation field, which is produced by this surface charge, is approximately zero. The total magnetic field inside the specimen is the sum of two terms: the externally imposed magnetic field $H_{e}$ and the demagnetisation field $H_{d}$ produced by the piece discontinuities,

(54) $$H=H_{e}+H_{d}.$$

Assuming an ideal current source excitation, the external field $H_{e}$ is proportional to the current and, hence, only consists of the basic harmonic. $H_{d}$, on the contrary, contains higher harmonic components, which are approximately zero at the coercive point. Therefore, we expect that the higher-harmonic part of *H*, comprising solely the $H_{d}$ contribution, will be nearly zero at the coercive point. In the case of voltage excitation, the coil current will also contain a small portion of higher harmonics, and $H_{e}$ will also comprise higher-harmonic components, which makes the location of $H_{c}$ less precise.

**Figure 9:**
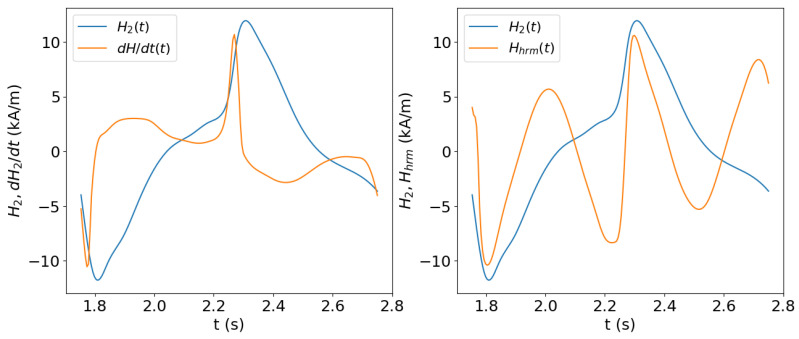
Calculated time dependence of the sample tangential field, $H_{2}\left(t\right)$, its time derivative, $dH_{2}/dt$, and the higher-harmonic component of $H_{2}\left(t\right)$ for the case of sinusoidal voltage in a magnetic circuit model (0D model). Plot reproduced from [59].

An illustration of the $d^{2}H/dt^{2}$, as well as the higher-harmonic part as a function of time, can be found in [59] and is reproduced in Figure 9. In the same plot, we also draw the variation of the higher-harmonic component of the magnetic field. The numerical results have been obtained by using the 0D approximation of the magnetic circuit presented in Section 4.2 for the closed magnetic circuit problem. One can readily observe the closeness of the two points. The numerical rresults were produced using the Ponomarev model for the sample with $H_{c}=2$ kA/m, $H_{0}=0.955$ kA/m, and $H_{m}=11.5$ kA/m. For more details of the considered setup, the reader is referred to the original paper [59].

**Figure 10:**
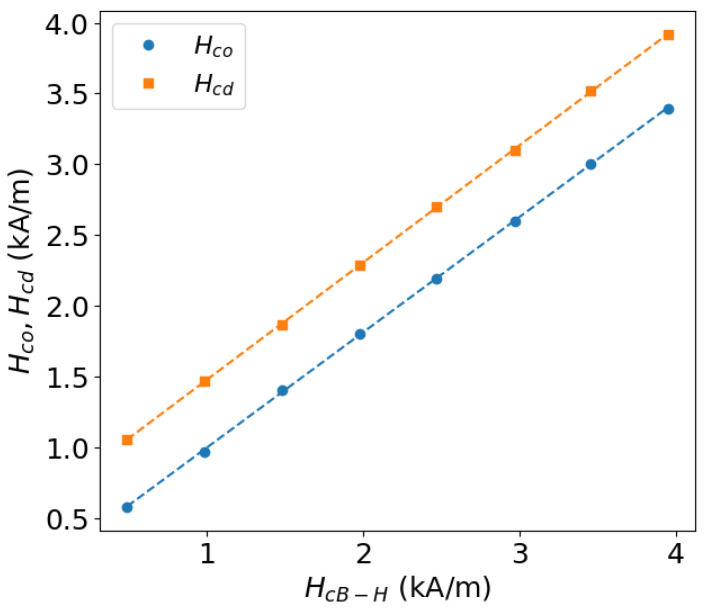
Correlation plots of the $H_{cd}$ and $H_{co}$ estimators vs. the real coercive field values $H_{cB-H}$. The dashed lines are obtained by linear fit to the data points. Plot reproduced from [59].

Figure 10 demonstrates the correlation of the coercive field approximations obtained by the two methods with the real value of the coercive field. Both estimators, i.e., the second-derivative zeros $H_{cd}$ and the cross-over points of the higher harmonics $H_{co}$, demonstrate a good correlation and, hence, can be used for the prediction of $H_{cB-H}$ from tangential field measurements after calibration.

### 4.3. Numerical and Statistical Analysis of Forward and Inverse MF Operator

The study of the correlation between physical observable quantities, model parameters, and material properties may provide a deeper physical insight into the model behaviour over a broad set of configurations. Nevertheless, data from experiments are often scarce, which limits the ability to draw meaningful conclusions on problems involving variability in the model factors to be evaluated (e.g., optimisation of a design, assessment of performances). The employment of numerical solvers has been demonstrated to enhance the analysis stage, based on experimental data, in terms of robustness, efficiency, and cost saving. Consequently, the integration of physics-based models with statistical methods has emerged as a prevailing methodology for addressing certain material characterisation problems.

In this context, the numerical modelling of the MF operator is pivotal in conducting advanced statistical studies on configuration that are pertinent to a specific problem. This includes highlighting potential unobserved or underestimated correlations between material parameters (p), setup parameters, and observables (y). As illustrated in Figure 1, the operators in (2) and (3) are essential in this regard if they can be numerically computed, enabling working with the constitution of datasets of simulations. Toward this end, the numerical counterpart of the total transfer MF operator can be modelled as

(55) MF:p⟼y

where, from now on, we refer to as the numerical operator. MF can then be utilised as an online operator to address optimisation tasks or to generate a synthetic dataset composed of finite number (e.g., *N*) of samples $D=\left\{\left(p_{0},y_{0}\right),\left(p_{1},y_{1}\right),\cdots ,\left(p_{N},y_{N}\right)\right\}$ to accelerate advanced numerical studies. In the latter case, based on a given dataset D, the numerical model itself is replaced by a suitable machine learning (ML) model (also called metamodel or surrogate model), based on a given dataset, with the aim of avoiding further calls to the physics-based model. Notwithstanding the reliance of MF rely on a physics-based or ML model, its potential for substantial exploitation can be employed to address optimisation problems, with the objective being the estimation of an unknown set of material parameters p based on some observed experimental measures $y_{exp}$ via the minimisation of a given cost function, for example,

(56) $$J\left(p\right)=\underset{p}{\mathrm{argmin}}{∥\mathrm{MF}\left(p\right)-y_{exp}∥}^{2}.$$

Furthermore, the application of the MF operator can be exploited for the computation of statistical indices, aiming to retrieve the impact of the hysteresis model parameter on the observables. That is, the numerical counterpart of the MF operator replaces the needed for the measures to come from the total transfer for computing the Global Sensitivity Analysis (GSA) indices such as the first-order Sobol’ ($S_{i}$), the δ-sensitivity measures ($\delta_{i}$), etc. [61].

As stated in (56), the inversion of p values based on the candidate observable $y_{exp}$ can be cast as an optimisation problem to be minimised iteratively. The inversion problem can be alternatively formulated as an inverse problem to be solved via ML methods. That is, one can define the numerical counterpart of the equivalent of the inverse total transfer $M^{-1}F^{-1}$ operator as

(57) $${\left(\mathrm{MF}\right)}^{-1}:y⟼p,$$

where the notation ${\left(\mathrm{MF}\right)}^{-1}$ indicates the explicit mapping performed by the operator, without distinguishing between the separated inversion of the $M^{-1}$ or $F^{-1}$, as outlined in (4). The selection of the most suitable ML schema is contingent upon the volume of data and the available computational resources, in addition to the anticipated outcomes. In principle, kernel machine-based algorithms, such as support vector machines, Gaussian process regressors, and kernel ridge regression, are suitable for addressing these tasks, in conjunction with deep neural network approaches.

## 5. Numerical Example

### 5.1. Harmonic Distortion Measurements in an Open-Circuit Configuration

The theoretical discussion of the previous sections can be better understood by a fully worked-out numerical example. The considered configuration is a basic open-circuit setup, based on the same working principle as that of the HACOM and the PropertyMon systems (both HACOM and PropertyMon systems are realisations of the open-circuit setup; the main differences from this example are the number and geometry of the inductors).

**Figure 11:**
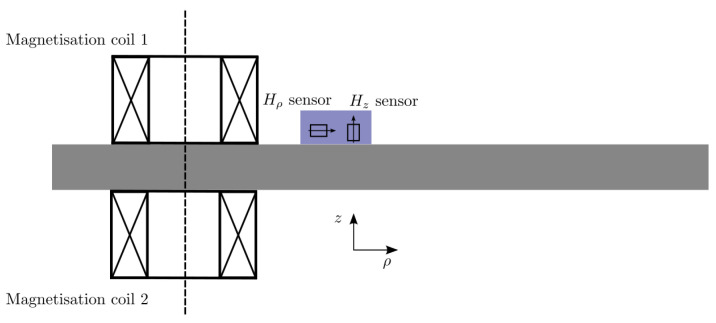
Open-circuit experimental setup for the measurement of the field harmonics.

The considered configuration is shown in Figure 11. The details of the geometry are given in [62] and are not repeated here for the sake of brevity. The studied material is a typical structural steel that can be practically considered as isotropic. The hysteresis model used in the study is the five-parameter Mel’gui model presented above. The hysteresis model is identified using the measured major hysteresis curve in a specialised characterisation experiment according to the standard procedure. A detailed description of the latter can be found in the same article [62].

The two inductors are fed with a sinusoidal voltage with 180° phase difference in order to enhance the tangential magnetic field component that passes through the plate and thereupon amplifies the material effect. Two excitation frequencies have been examined: 10 Hz and 20 Hz. Note that the frequency must be kept low in order to minimise the eddy-current effects, which hinder the magnetic field penetration in the interior of the specimen.

The field problem is solved numerically using the finite integration technique (FIT). A major difficulty when treating material with hysteresis consists of the multi-branch behaviour of the material curve at the reversal points of the feed current, which may destabilise the numerical solver. A further challenge stems from the low-field inner loops, for which most parametric models (including the Mel’gui model considered herein) behave poorly. Both issues can be remedied by considering the hysteresis law in an augmented parametric space, as explained in [53,62].

**Figure 12:**
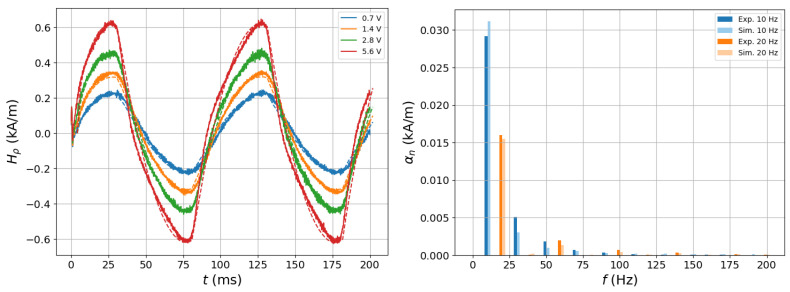
(**Left**) Comparison of the measured time signal of the tangential magnetic field at a specific observation point with the corresponding simulation curves. One observes the deviation from the harmonic behaviour with increasing excitation voltage. The measurement frequency is 10 Hz. (**Right**) Measured vs. simulated spectra for the maximum signal at the same observation point and for both frequencies.

Figure 12 shows a comparison between the measured and simulated field response for a given position of the field sensor. In the left plot, there are the measured signals of the tangential magnetic field for four different excitation voltages. It is easily observed that the signal deviates from the sinusoidal form with increasing field intensity. The corresponding spectra obtained for the maximum level of excitation at both frequencies are shown in the right-hand plot.

It is interesting to note the fast decrease in the amplitude with increasing harmonic index. This means that the essential (and exploitable) information must be sought in the first harmonics since higher-harmonics are expected to be under the noise threshold. Obviously, the higher the excitation signal, the better the signal-to-noise ratio that should be attained; however, the aforementioned steep decrease should be kept in mind.

### 5.2. Correlation with Hysteresis Parameters

Once the numerical model of the problem is established and validated, we can turn our attention to the relation of the material parameters with the measurement observables. In this sense, we exploit simulations based on a design of experiment (DoE), accounting for the measurement setup parametrisation in terms of excitation frequency, voltage excitations, and the Mel’gui model parameters. The variations in the Mel’gui parameters used for the establishment of the DoE are described in Table 3.

The main purpose of the analysis is to verify how Mel’gui parameters correlate with the harmonic amplitudes for the different measurement frequencies and the different excitation levels. Toward this end, the DoE given in Table 3 is scanned to generate a dataset composed of 766 samples containing a time signal of 100 timesteps, associated with the tangential and normal field components. By applying the Fourier transform to the output signals, the measurement spectrum is obtained, from which only the first three harmonics having the highest amplitudes (and, thus, being less impacted by noise) are considered.

For the formal analysis, the Pearson’s and Spearman’s correlation coefficients have been employed. The former one postulates an ‘almost’ linear dependence between analysed variables, whereas the latter exploits the monotonicity in the analysed factors [63]. The Pearson’s correlation coefficient is defined as

$$r_{Pearson}\frac{\sum_{i=1}^{n}\left(x_{i}-\bar{x}\right)\left(y_{i}-\bar{y}\right)}{\sqrt{\sum_{i=1}^{n}{\left(x_{i}-\bar{x}\right)}^{2}}\sqrt{\sum_{i=1}^{n}{\left(y_{i}-\bar{y}\right)}^{2}}}$$

with the subscript *i* indicating the *i*-th sample and $\bar{x}$ and $\bar{y}$ referring to the mean values of *x* and *y*, respectively. The Spearman’s correlation coefficient is defined as

$$r_{Spaerman}=\frac{\mathrm{cov}\left(R\left[X\right],R\left[Y\right]\right)}{\sigma_{R\left[X\right]}\sigma_{R\left[Y\right]}}$$

where $R\left[X\right]$ and $R\left[Y\right]$ represent the rank variables. In this work, the samples $x_{i}$, $y_{i}$ and the stochastic variables *X* and *Y* are defined based on the Mel’gui model factors and the observable, which are considered such that X=Y=$\left\{M_{s},H_{c},M_{r},M_{c},\chi_{in},V\right\}$, *V* is the excitation voltage.

**Figure 13:**
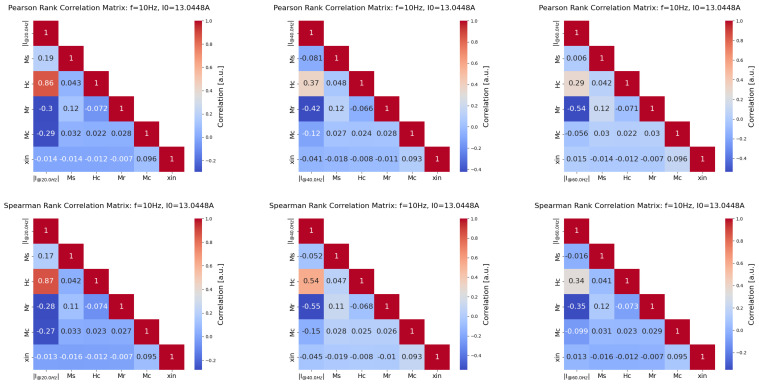
Pearson’s (**upper**) vs. Spearman’s (**lower**) correlation coefficient, calculated between the material parameters and the measured tangential field at 10 Hz and the highest excitation voltage. From left to right: field amplitude of 1st, 3rd, and 5th harmonics.

Toward this end, we discussed the results obtained from the point of view of correlations and qualitative plots in order to highlight the most meaningful outcomes observed. In Figure 13, we analysed the Spearman’s and Pearson’s correlations between Mel’gui model factors and the amplitude of the the spectrum at the first three harmonics. Overall, we notice that Pearson’s and Spearman’s correlation indexes agree with each other, regardless of the components considered, as shown in the first columns of each plot. Furthermore, regardless of the harmonic considered, $H_{c}$ and $M_{r}$ are the two factors that correlate more with the amplitude of the spectral components considered, whereas the weakest correlation across the harmonics is always between $\chi_{in}$ and $M_{s}$.

Furthermore, it has been observed that $\chi_{in}$ does not correlate with the amplitude of the spectrum for the investigated frequencies and, thus, could be likely discharged from the model parameters, with minor changes in the measured quantities. It has been also observed that one can take advantage of the correlation analysis of Mel’gui parameters to choose the most-suitable harmonic contents to be analysed in order to possibly retrieve the Mel’gui model parameters when the inversion of the hysteresis parameters is targeted [64].

### 5.3. Sensitivity Analysis of Hysteresis Parameters Based on Harmonic Distortion Measurements

Sensitivity analysis is a statistical methodology that aims to assess the ‘sensitivity’ of one or multiple observables against the variation in a set of factors that are supposed to have an impact on these observables. The sensitivity analysis estimation enables one to identify and/or rank how a specific parameter leads to changes in the observables. In statistics, multiple methods have been developed in order to perform sensitivity analysis studies; the most advanced one requires one to establish a meaningful statistical set in order to draw meaningful and robust conclusions [61]. For the sake of brevity, it is worth mentioning that, based on the state-of-the-art literature, we consider in the following the use of the Sobol’ and δ-sensitivity measurement GSA methods, which are well-established in the applied statistics community to assess the uncertainties in complex numerical models [61]. One can show that the Sobol’ indices’ calculation, in the case of the estimation of the separated effects of the *i*-th set of a factor onto the observable—the so-called first-order Sobol’ indices—representing the effects of the *i*-th factor on the output, is defined as

$$S_{i}=\frac{V_{i}}{Var\left({∥\mathrm{MF}\left(p\right)∥}_{\infty }\right)},$$

where ${∥\cdot ∥}_{\infty }$ stands for the maximum value of the ML-based operator, evaluated on the samples of a given set of factors p, $V_{i}=Var_{p∼i}\left(E_{p∼i}\left({∥\mathrm{MF}\left(p\right)∥}_{\infty }|\left(p_{i}\right)\right)\right)$, with $E_{x∼i}$ representing the expectation and $Var_{p∼i}$ representing the variance, calculated without the contribution of the *i*th factor [65]. Sobol’ indices are suitable measurements of the sensitivity based on the variance decompositions. In certain classes of problems, as a function of the observable considered (e.g., the amplitude of specific harmonic components, the amplitude of the measured signal), variance decomposition may not the most suitable approach; it is then interesting to perform GSA based on moment-independent indices like the δ-sensitivity measures for the *i*-th factor, which are defined as

$$\delta_{i}=0.5E_{Pi}\left[s\left(P_{i}\right)\right],$$

with $E_{Pi}\left[s\left(P_{i}\right)\right]$ referred to as the inner statistic or separation, which represents the surface of the intersection between the conditional and unconditional probability densities of the model output ${∥\mathrm{MF}\left(p_{i}\right)∥}_{\infty }$ obtained for the *i*th factor value [66].

GSA indices rely on the calculation of scalar quantities, which are supposed to capture the overall variability of the signal, making them suitable for quantifying the impact of the hysteresis parameter model on the harmonic distortion measurements.

**Figure 14:**
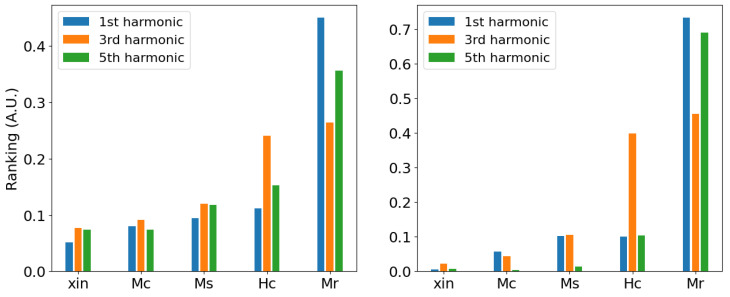
Global sensitivity analysis indices of Mel’gui parameters for the three first harmonics calculated between the material parameters and the measured tangential field at 10 Hz and the highest excitation voltage. (**Left**) δ-sensitivity measures. (**Right**) First-order Sobol’ indices.

Looking at Figure 14, and comparing these results with the correlation plots shown in the previous section, one can notice that the ranking obtained via GSA is aligned with the results. Furthermore, since GSA calculation are based on the use of a metamodel, the demanding statistical calculation can be computed in a efficient way, leading to a factor ranking calculation that is less sensitive to the dataset used. On top of that, GSA enables the estimations of the uncertainties associated with the ranking too. In view of the inversion procedure, the analysis of GSA can qualitatively provide some insight on the ill-posedness of the inverse problem by analysing the ranking results.

### 5.4. Prediction of the Material Parameters via Direct Inversion of MF Operator

The reliable and fast inversion of the MF operator is an open and ongoing research field that may further enhance the analysis capability based on measured data. Regardless of the inversion procedure used, i.e., iterative (56) or non-iterative (57) ones, one postulates the access to a set of possible solutions in order to retrieve the actual parameters from the observable. Considering non-iterative inversion, which mainly relies on the use of ML algorithms trained in a supervised framework, one can define a regressor operator ${\left(\mathrm{MF}\right)}^{-1}$, as defined in (57) based on a specific ML model. The choice of the algorithm typically depends on the number of samples available for training the ML model as well as the cardinality of the measures and the factors to be estimated. In the context of material parameter estimation, based on the Mel’gui model, the use of kernel machines, like the Gaussian process regressor, support vector machine, etc., or effective neural network architecture can be considered.

**Figure 15:**
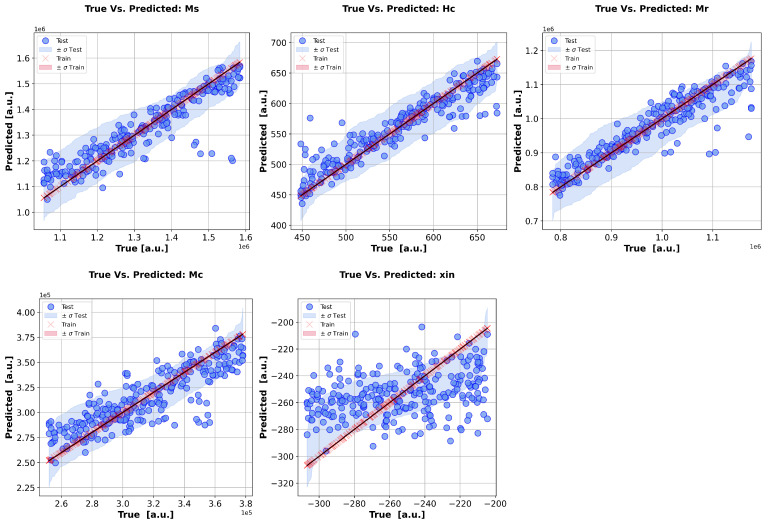
Inversion results for the Mel’gui parameters based on Gaussian process regressor of ${\left(\mathrm{MF}\right)}^{-1}$ operator. From left to right: Ms, Hc, Mr, Mc, and χin predictions on training and test sets are provided, along with the estimation of the associated uncertainties.

Having examined the correlations between the hysteresis parameters (inputs) and field measurements (outputs), we now shift the focus to the direct output inversion by the numerical calculation of the ${\left(\mathrm{MF}\right)}^{-1}$ operator. Given the ill-posedness of this inversion operator, we restrict the study to a DoE, which consists of a Latin hypercube, with each side spanning a ±20% interval of the nominal parameter values (with the term nominal meaning the Mel’gui parameters obtained via identification). Based on this dataset, we consider a set of samples, i.e., the so-called training set, from which the ML is based, such that, in the case of a Gaussian process regressor, is given as ${\left(\mathrm{MF}\right)}^{-1}\left\{y\right\}=\sum_{i}w_{i}\left(y\right)p_{i}$ [64]. Thus, to compute, by inversion, the model parameters ${\hat{p}}_{exp}$ (posterior mean) associated with an experimental measure $y_{exp}$ are given as ${\hat{p}}_{exp}=K\left(y_{exp},y\right)K{\left(y,y\right)}^{-1}p$, with K(·,·) representing a suitable covariance matrix. The uncertainty associated with the predictions can be straightforwardly computed as $\sum_{{\hat{p}}_{exp}}=K\left(y_{exp},y_{\exp}\right)-K\left(y_{exp},y\right)K{\left(y,y\right)}^{-1}K\left(y,y_{\exp}\right)$. The inversion results for the hysteresis parameters are graphically depicted in Figure 15. Each point in these plots stands for the inverted input (hysteresis parameter) of a synthetic field signal, randomly chosen from the DoE. The predictions of the input parameters are compared against the true input parameters used for the calculation of the synthetic signal. Note that, the closer to the diagonal that the points lie, the better the predictions. The shadowed area around the parameters highlights the uncertainties (±a standard deviation) associated with the predictions. A cross-analysis of Figure 14 and Figure 15 readily highlights the fact that the less-sensitive parameters have a harder inversion task. Such results also highlight the fact that the analysis of GSA indecision via a forward metamodel based on single harmonic contents in observable signals can be considered in order to qualitatively foresee the ‘degree’ of the ill-posedness of the inversion problem.

**Figure 16:**
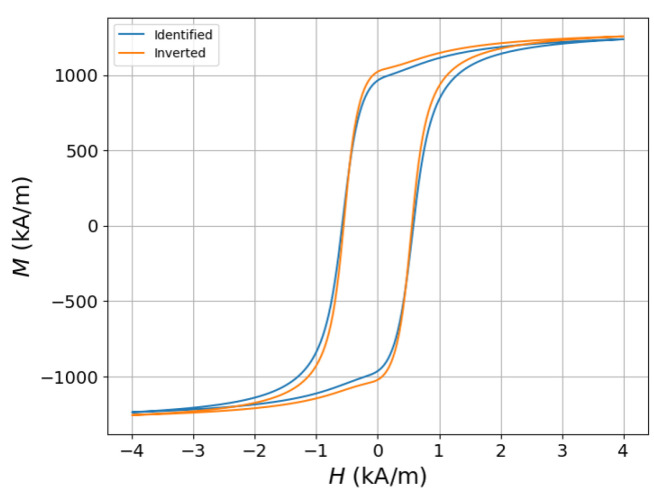
Comparison of the inversion results for the B(H) curve, with the identified curve obtained by the experimental data.

In Figure 16, the hysteresis curve obtained upon the inversion of the experimental data in [62] is compared against the identified hysteresis curve of the material. Despite the preliminary nature of these results, they demonstrate the potential of moving a step further than the usual calibration curves used by the majority of state-of-the-art equipment and performing direct predictions of the hysteresis parameters based on open-circuit measurements. Similar conclusions are presented in [64], where the numerical inversion of impulse response measurements have been used to retrieve the material parameters.

## 6. Correlation with the Microstructure and the Mechanical Properties

Upto this point, the ferromagnetic materials have been characterised via the parameters of the hysteresis models (i.e., its magnetic properties), which provide only an indirect link with the material microstructure. For a nondestructive assessment of the latter, however, a more direct link is necessary.

Still, the dependence of the magnetic properties on the microstructure is an extremely complex problem, which has been a subject of intensive study since the 1940s, with the seminal contributions of Kersten and others, with a general quantitative relation still missing. Nevertheless, notable progress has been made in the understanding of the main mechanisms that influence the wall movements, which has resulted in several useful empirical relations. Among the most-important features that have an impact on the magnetic and mechanical properties, one can mention the solid-solution proportion, the dislocation density, the grain size, the precipitates and/or second phase domains, and the texture.

Dislocations act as impediments to Bloch wall movements. The majority of studies converge to the conclusion that the coercive field $H_{c}$ is proportional to the square root of the dislocation density ρ [67]:

(58) $$H_{c}∼\sqrt{\rho }.$$

Grain boundaries represent two-dimensional lattice defects, which also act as domain wall impediments. There is again general agreement on the mathematical expression of this dependence, which admits the following inverse exponential form [67,68,69,70,71,72]:

(59) $$H_{c}=A_{0}+\frac{A_{1}}{d^{n}}$$

where $A_{0},A_{1}$ are constants, *d* stands for the mean grain size, and *n* is an exponent whose value lies in the interval [0.5,2]. One must note that there is no general consensus on the exact value of the *n* exponent, with the majority of researchers tending to n=1. Regardless of the *n* value, (59) expresses an inverse dependence on the grain size, that is, the smaller the grain, the magnetically harder the material, which is consistent with the physics.

Sablik and Dupré used the combined form of the above two relations, namely,

(60) $$H_{c}\left(\rho ,d\right)=\left(A_{0}+\frac{A_{1}}{d^{n}}\right)\sqrt{\rho }.$$

with $A_{0}$, $A_{1}$, and *n* being determined upon fitting with experimental data [71,72].

The effect of the precipitates is more complicated since the total effect depends on several factors like the type, quantity, size, distribution, and hardness of the particles. It is, therefore, harder to obtain a simple quantitative general expression. Existing attempts are based on the foreign-particle theory, which roots back to Kersten but is not considered here [73,74]. Second-phase aggregates are also expected to have a similar effect.

Finally, the existence of texture is responsible for anisotropy and, hence, can be indirectly taken into account by assuming the directional dependence of the magnetic characteristics.

The yield-strength dependence on the microstructure shows similar trends with the coercive field, which is plausible since the same lattice irregularities responsible for the wall-pinning during magnetisation also act as impediments to the lattice layer creep, which is the main mechanism of plasticity. In particular, the empirical expression that relates the yield strength with the microstructure characteristics admits the general form

(61) $$\sigma_{y}=\sigma_{0}+\sigma_{ss}+\sigma_{\rho }+\sigma_{d}+\sigma_{p}$$

where $\sigma_{0}$ is for the Peierls–Nabarro stress (the tension to move dislocations at T=0 K), $\sigma_{ss}$ expresses the influence of the solid-solution atoms (i.e., substitution atoms like Si, Mn, or P or interstitial atoms like C or N), $\sigma_{\rho }$ stands for the dislocation effect, $\sigma_{d}$ is the influence of the grain side, and $\sigma_{p}$ describes the contribution of the precipitates. $\sigma_{\rho }$ and $\sigma_{d}$ follow similar laws with the corresponding ones for the coercive field, namely,

(62) $$\sigma_{\rho }∼\sqrt{\rho }$$

and

(63) $$\sigma_{d}∼\frac{1}{\rho^{1/2}}$$

known as Hall–Petch and Taylor expressions, respectively. The precipitate effect is shown to be analogous to the mean distance between precipitates.

**Figure 17:**
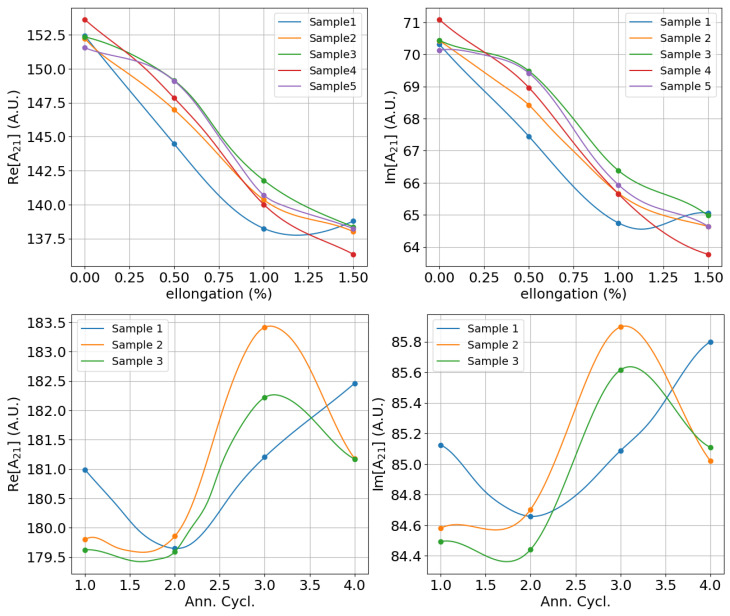
First harmonic as a function of (**upper**) grain elongation and (**lower**) annealing time, expressed in number of annealing cycles. Measurements have been carried out using the HACOM device. Plots reproduce with permission from [26] (all values have been rescaled by a factor of $10^{3}$ for better visibility).

The experimental demonstration of the dependence between material microstructure and harmonic measurements is not a simple task since one needs to perform measurement campaigns using sets of different, well-characterised microstructures. Such systematic studies can be found in a number of reports from past Research Fund of Coal and Steel (RFCS) projects [25,26,30]. An example of the variation of basic harmonic as a function of grain elongation and annealing time for different cold-rolled steel samples of DX56D-Z grade is shown in Figure 17.

**Figure 18:**
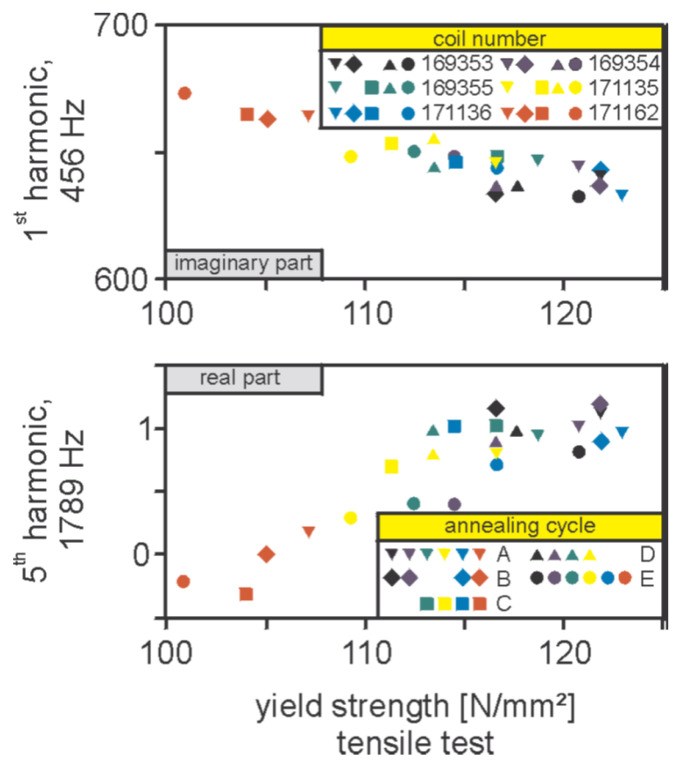
First and fifth harmonic measurements vs. the yield strength value $R_{p0.2}$. Different markers correspond to IF samples with slightly different treatment (sources [25]).

The similarity between (58), (59) and (62), (59) implies a direct relation between $H_{c}$ and $\sigma_{y}$. This is indeed confirmed by the correlation of harmonic distortion measurements with destructive measurements of the yield strength. An example of such a correlation is given in Figure 18, where the HACOM measurements of the first and fifth harmonics for a number of samples of conventional IF are compared against destructive measurements of $R_{p0.2}$.

## 7. Conclusions

The harmonic distortion measurement of the magnetic field signal in ferromagnetic materials is a flexible non-destructive technique, which offers rich information about the material’s magnetic properties. The main features of these properties can be retrieved by a basic analysis of the spectral information, notably, the harmonic ratio and the harmonic distortion identifier, which is the main analysis approach used by available commercial systems (PropertMon, 3MA). In a second step, this information, combined with an appropriately calibrated regressor (using well-characterised reference specimens), can be applied for the estimation of mechanical and other technological material properties. A regression example is shown in Figure 18, where a clear relation between the harmonic content and the yield strength is demonstrated.

The recent advances in the simulation techniques and the rapidly transforming landscape of signal processing and inversion algorithms, including machine learning, aided by the tremendous progress in hardware architecture, pave new directions for the processing of measurement data. The results in Section 5.4 demonstrate the possibility to retrieve the hysteresis curves via the numerical inversion of harmonic distortion measurements. The quality of these predictions is expected to be further enhanced when enriched with other indicators (like the incremental permeability) in comprehensive data-fusion models, which can also benefit from the new possibilities offered by tools like artificial neural networks. For example, 3MA, already offers such models, and a lot of research is currently conducted in this direction.

New exciting perspectives involving multiscale simulations, which are capable of taking features of the grain structure directly into account, give hope for a new generation of powerful tools that will provide estimations of material features that are presently accessible only via microscopic methods [30,75].

## Figures and Tables

**Table 1:** Summary of the different parametric models considered for the hysteresis description. Note that $M_{R}$, not the form parameter $H_{0}$ of the definition relation, is chosen as the third parameter in the Ponomarev model. Given that the two parameters are related via (19), the former has been preferred for conformity with the other models.

Model	Parameters	Domain of Description
Langevin	$M_{s},\chi_{0}$	high-field
Backlash	$H_{c},\chi_{c}$	close to $H_{c}$
Ponomarev	$M_{s},H_{c},M_{R}$	irreversible
Mel’gui	$M_{s},H_{c},M_{R},\chi_{0},M_{c}$	irreversible, reversible

**Table 2:** Ponomarev’s model parameters for the studied IF steel grade obtained by identification.

Parameter	Value
$M_{s}$	721.694 kA/m
$H_{c}$	0.751 kA/m
$H_{0}$	0.326 kA/m

**Table 3:** Design of experiments used to perform the numerical studies based on the industry-like setup presented in [62].

Mel’gui Model Parameters	Intervals	Sampling Strategy
$M_{s}$	[1.056,1.584] MA/m	Uniform
$H_{c}$	[448.408,672.612] A/m	Uniform
$M_{r}$	[0.784,1.176] MA/m	Uniform
$M_{c}$	[0.252,0.378] MA/m	Uniform
$\chi_{in}$	[−306.828,−204.552]	Uniform

## Data Availability

The raw data supporting the conclusions of this article will be made available by the authors on request.

## Appendix A. Derivation of the Ponomarev Model

In this appendix we try to derive the Ponomarev formula (17) for major and minor symmetrical loops, i.e., loops emanating from the first magnetisation curve, by first principles and using the well-established symmetry properties of the hysteresis curves.

Following [55], in an Ising system with supercritical disorder, the differential susceptibility in the proximity of the coercive field $H_{c}$ can be approximated to a very good extent by a Lorentzian function:

(A1) $$\chi \left(H\right)=\frac{\chi_{c}}{1+{\left(\frac{H-H_{c}}{\gamma }\right)}^{2}}.$$

where $\chi_{c}$ is the differential susceptibility at the coercive field and γ is a parameter that has magnetic field units and is linked to the material disorder. In order to conform with the symbols in the remainder of the text, we write, from now on, $\gamma =H_{0}$.

Before moving to the derivation of the relation, we make two additional postulates:

1. We assume that a polycrystalline steel with a uniaxial excitation can be coarsely approximated by an Ising system;
2. We accept that (A1) is generally valid for the entire region of interest and not only around the coercive field.

Let us now assume that we are located at an arbitrary point of the negative part of the virgin curve ($-H_{r},-M_{r}$), with $H_{r},M_{r}>0$, and we start to increase the external magnetic field *H*. The magnetisation will then depart from the virgin curve, following the ascending branch of the minor loop that corresponds to the ($-H_{r},-M_{r}$) reversal point. The magnetisation curve at *H* is obtained by integrating (A1):

(A2) $$\begin{array}{c} M\left(H\right)=\chi_{c}\gamma \arctan\left(\frac{H-H_{c}}{H_{0}}\right)+c \end{array}$$

where *c* is an integration constant. The integration constant can be eliminated by applying (A2) at the departure point $\left(-H_{r},-M_{r}\right)$, so (A2) becomes

(A3) $$\begin{array}{c} M\left(H\right)=\chi_{c}\gamma \left[\arctan\left(\frac{H-H_{c}}{H_{0}}\right)+\arctan\left(\frac{H_{r}+H_{c}}{H_{0}}\right)\right]-M_{r}. \end{array}$$

The ascending branch will again meet the virgin curve at the positive reversal point, which is owed to the point-symmetry property of hysteresis being located at ($H_{r},M_{r}$). Again applying (A3), we can determine $M_{r}$ in terms of the Lorentzian parameters and the magnetic field value at the reversal point $H_{r}$, namely,

(A4) $$\begin{array}{c} M_{r}=\frac{\chi_{c}H_{0}}{2}\left[\arctan\left(\frac{H_{r}-H_{c}}{H_{0}}\right)+\arctan\left(\frac{H_{r}+H_{c}}{H_{0}}\right)\right]. \end{array}$$

Due to the fact that the ensemble of the reversal points are located on the virgin curve, (A4) can be considered as the expression of the latter.

The descending branch can now be easily determined by once more applying the point-symmetry property. We thus arrive at the general expression

(A5) $$\begin{array}{c} M^{\pm }\left(H\right)=\chi_{c}H_{0}\arctan\left(\frac{H\mp H_{c}}{H_{0}}\right)\pm M_{r}. \end{array}$$

with the upper sign standing for the ascending branch and the lower for the descending.

The asymptotic value of (A4) for large magnetic field values should be the saturation magnetisation, from which we obtain the interesting relation

(A6) $$\chi_{c}H_{0}=\frac{2M_{s}}{\pi }$$

which relates the disorder parameter $H_{0}$ with the saturation value. Substituting in (A5) and (A4), we arrive at the relations

(A7) $$M^{\pm }\left(H\right)=\frac{2M_{s}}{\pi }\arctan\left[\frac{\pi }{2}\left(\frac{H\mp H_{c}}{H_{0}}\right)\right]\pm \frac{2M_{s}}{\pi }r_{m}$$

and

(A8) $$\begin{array}{c} r_{m}=\frac{1}{2}\left\{\arctan\left[\frac{\pi }{2}\left(\frac{H_{r}-H_{c}}{H_{0}}\right)\right]+\arctan\left[\frac{\pi }{2}\left(\frac{H_{r}+H_{c}}{H_{0}}\right)\right]\right\} \end{array}$$

which reproduce the original Ponomarev formulation.

## Appendix B. Spectral Decomposition of the Ponomarev Expression

We are interested in deriving approximate relations for the magnetisation spectrum involving explicit expressions of the model parameter. The starting point is the Ponomarev expression, which is repeated here for convenience:

(A9) $$M\left(H\right)=\frac{2M_{s}}{\pi }\arctan\left(\frac{H-sH_{c}}{H_{0}}\right)+s\frac{2M_{s}}{\pi }r_{m}$$

with *s* being a sign function denoting the working branch, namely,

(A10) $$s=\mathrm{sgn}(\frac{dH}{dt}).$$

Assuming a sinusoidal magnetic field, $H=H_{m}\sin\omega_{0}t$ can be written as

(A11) $$M\left(H\right)=\frac{2M_{s}}{\pi }\arctan\left[\frac{H_{m}\sin\omega_{0}t+s\left(t\right)H_{c}}{H_{0}}\right]+s\frac{2M_{s}}{\pi }r_{m}$$

where

(A12) $$s\left(t\right)=\frac{\cos\omega_{0}t}{|\cos\omega_{0}t|}.$$

Note that the field amplitude is not a material parameter and it is not of interest us as such, yet it has an impact on the magnetisation function. We set $a=H_{m}/H_{0}$; it is reasonable to assume that it remains constant throughout our study since it expresses how deeply in the saturation domain we arrive for a given hysteresis shape. Furthermore, for large $H_{m}$, we do not expect a dramatic form change in the signals. Thereupon, *a* can be kept out of our analysis.

The development of the arctan function of (A11) in Taylor series with respect to $s\left(t\right)H_{c}/H0$ yields

(A13) $$\begin{array}{cc} M(t) & \approx \frac{2M_{s}}{\pi }\left[\arctan\left(a\sin\omega_{0}t\right)+r_{m}s\left(t\right)\right]-\frac{2M_{s}}{\pi }\sum_{k=1}^{\infty }c_{k}\left(t\right)s^{k}\left(t\right){\left(\frac{H_{c}}{H_{0}}\right)}^{k}. \end{array}$$

The series converges very fast for $H_{c}<H_{0}$. In the opposite case, the convergence is very slow in the interval $-H_{c}\leq a\sin\omega_{0}t\leq H_{c}$. This can be improved by choosing another development point for the series, i.e., at $H_{c}/H_{0}=1$. To keep the discussion simple, we do not enter into more details about assuming an adequate convergence at any point in the hysteresis curves. Under this condition, we only need to consider the lowest terms of the series. The coefficients up to the fifth order read

(A14) $$\begin{array}{cc} c_{1}\left(t\right) & =\chi_{0}\left(t\right) \end{array}$$

(A15) $$\begin{array}{cc} c_{2}\left(t\right) & =\chi_{0}^{2}\left(t\right)a\sin\omega_{0}t \end{array}$$

(A16) $$\begin{array}{cc} c_{3}\left(t\right) & =\chi_{0}^{3}\left(t\right)\left(3a^{2}\sin^{2}\omega_{0}t-1\right) \end{array}$$

(A17) $$\begin{array}{cc} c_{4}\left(t\right) & =\chi_{0}^{4}\left(t\right)a\sin\omega_{0}t\left(a^{2}\sin^{2}\omega_{0}t-1\right) \end{array}$$

(A18) $$\begin{array}{cc} c_{5}\left(t\right) & =\chi_{0}^{5}\left(t\right)\left[4a^{4}\sin^{4}\omega_{0}t-12a^{2}\sin^{2}\omega_{0}t-{\left(a^{2}\sin^{2}\omega_{0}t+1\right)}^{2}\right] \end{array}$$

with

(A19) $$\chi_{0}\left(t\right)=\frac{1}{a^{2}\sin^{2}\omega_{0}t+1}.$$

It can be easily shown that M(t) is an odd-function periodic function, i.e., it fulfills the anti-symmetry property

(A20) M(t+T)=−M(t)

where $T=2\pi \omega_{0}$ is the function period. This is trivial to show for the first term in (A11) as well as for the series terms with *k* = odd since s(t+T)=s(t) and $c_{k}\left(t\right)$ consists of even powers of the sine function. For the even terms, $s{\left(t\right)}^{2}=1$ and $c_{k}=f_{e}\left(t\right)\sin\omega_{0}t$, with $f_{e}\left(t\right)$ even. Hence, their product is an odd function.

The Fourier series of (A11) involves only odd harmonics, owing to the anti-symmetry of the time function, whose expression reads

(A21) $$M_{2n+1}\approx \frac{2M_{s}}{\pi }\sum_{k=0}^{5}c_{k,n}{\left(\frac{H_{c}}{H_{0}}\right)}^{k}e^{-i\left(2n+1\right)\omega_{0}t}$$

for n=0,1,…, with

(A22) $$c_{0,n}=\frac{1}{T}\int_{0}^{T}\left[\arctan\left(a\sin\omega_{0}t\right)+r_{m}s\left(t\right)\right]e^{-in\omega_{0}t}dt$$

and

(A23) $$c_{k,n}=\frac{1}{T}\int_{0}^{T}s^{k}\left(t\right)c_{k}\left(t\right)e^{-in\omega_{0}t}dt.$$
